# Supplementary material for: Ecological Significance of a Novel Nitrogen Fixation Mechanism in the Wax Scale Insect Ericerus pela
Source: Insects. 2025 Aug 13;16(8):836. doi: 10.3390/insects16080836 (PMC12386322; doi:10.3390/insects16080836)
Supplement: Supplementary file 1 [file insects-16-00836-s001.zip › Figure S3.pdf]

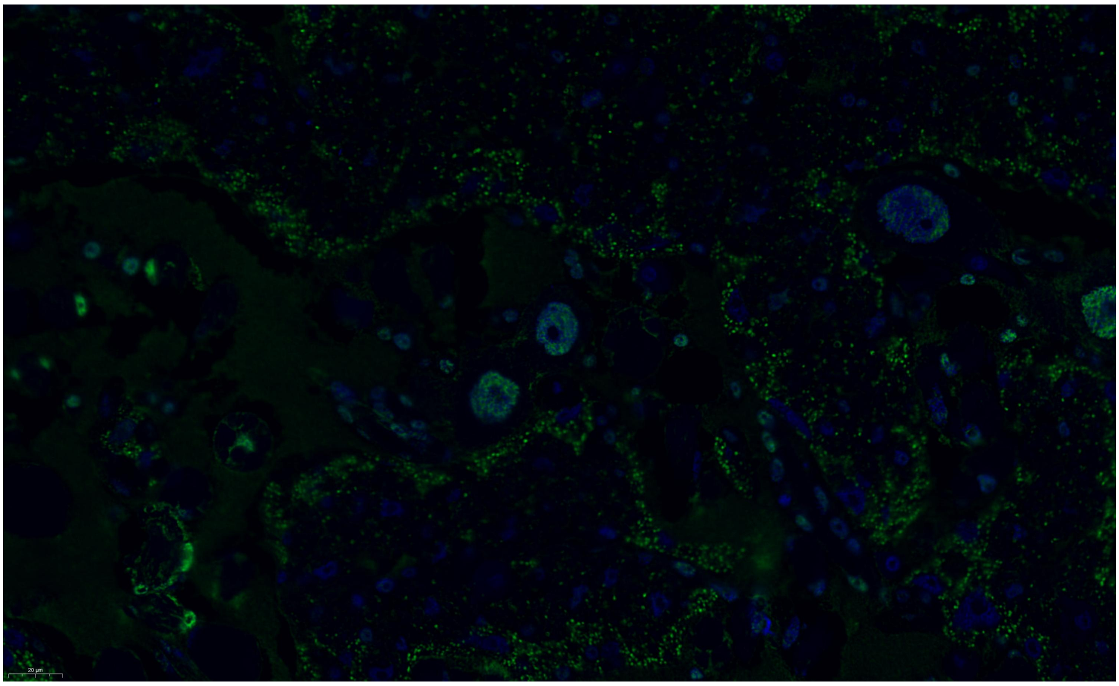

Figure 2. Nitrogen-fixing bacteria in *E. pela*

C. Immunofluorescence localization demonstrating the presence of *Rhizobiales* and *Methylbacterium* within fat body cells.

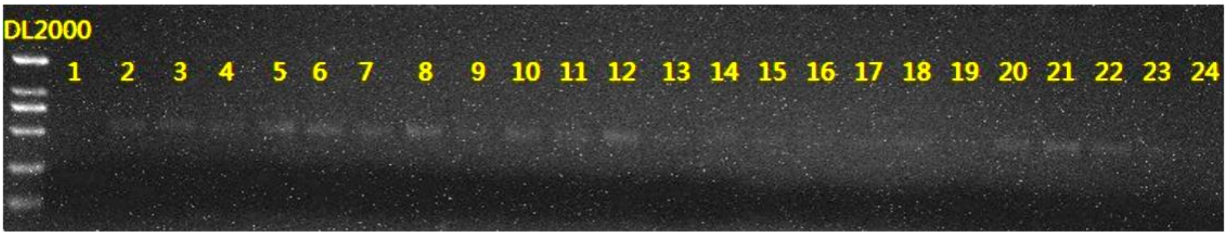

Figure 2. Nitrogen-fixing bacteria in *E. pela*

D. The *nif*H gene was detected in all developmental stages of *E. pela.*

(1-3: eggs; 4-6: female 1st instar nymphs; 7-9: male 1st instar nymphs; 10-12: female 2nd instar nymphs; 13-15: male 2nd instar nymphs; 16-18: pupae; 19-21: female adults;: 22-24: male adults)
