# Supplementary material for: Ecological Significance of a Novel Nitrogen Fixation Mechanism in the Wax Scale Insect Ericerus pela
Source: Insects. 2025 Aug 13;16(8):836. doi: 10.3390/insects16080836 (PMC12386322; doi:10.3390/insects16080836)
Supplement: Supplementary file 1 [file insects-16-00836-s001.zip › Figures S1 and S2.pdf]

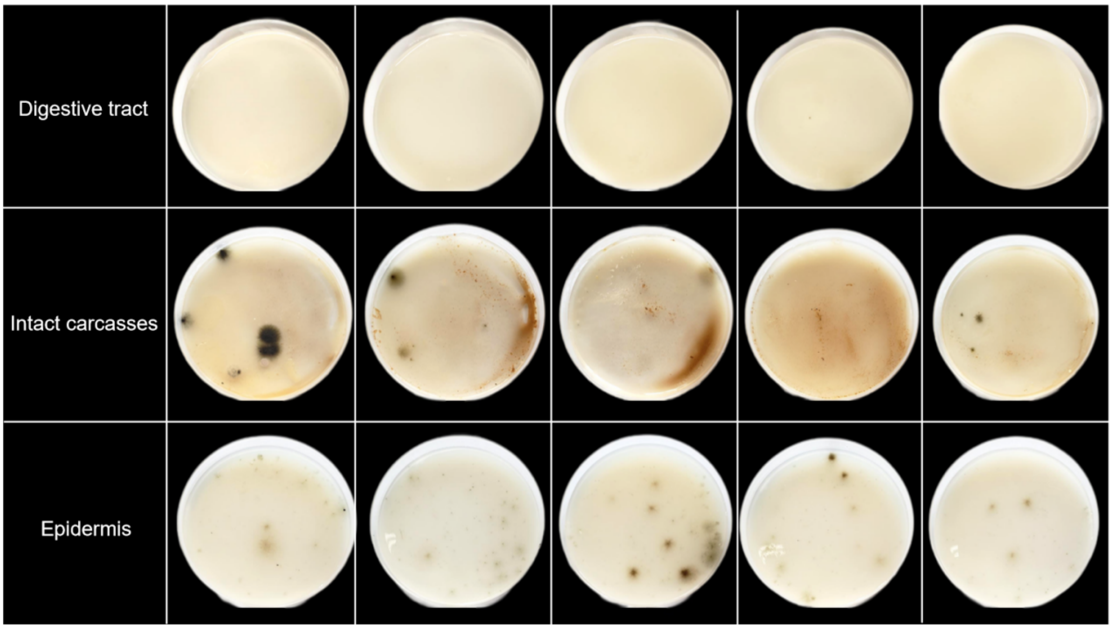

Figure S1. microorganism growth of different tissues of *E. pela* on nitrogen-free culture-medium

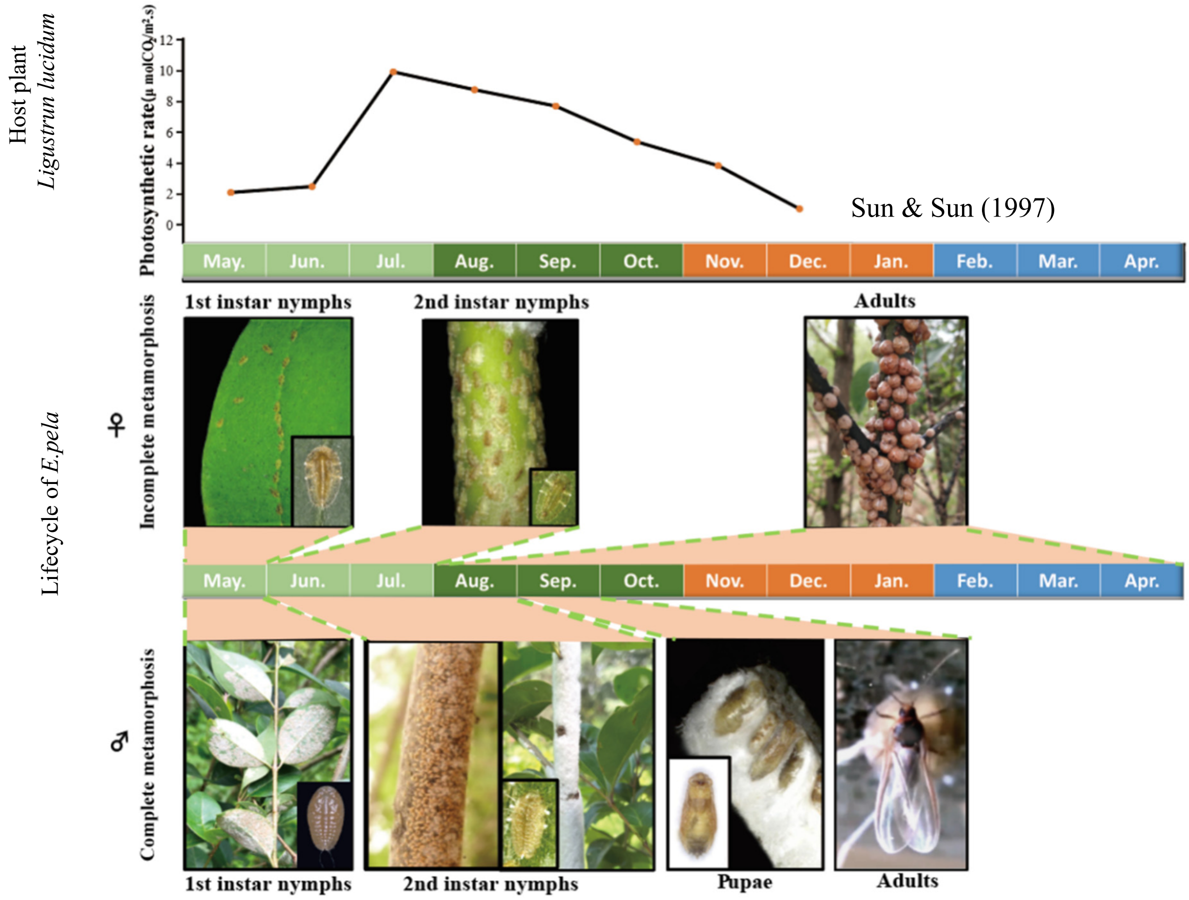

Figure S2. Lifecycle of *E. pela* and photosynthesis of its host plant

In the lifecycle of *E.pela*, female is incomplete metamorphosis, undergo two instars nymphs and adult. Male is complete metamorphosis, undergo two instar nymphs,2nd nymph and pupae and adult. 1st instar female nymphs live on the front leaf, 1st instar male nymphs live on the back leaf, after about four weeks, 2nd instar nymphs transferred to branch, female nymphs live on shoot and developed adult, male live on branch and secreted wax to cover body, developed pupae in wax and adult fly out after eclosion in wax. Photosynthesis of host plant *Ligustrun lucidum* was cited in Sun & Sun (1997).
